# Deoxynivalenol modulated mucin expression and proinflammatory cytokine production, affecting susceptibility to enteroinvasive *Escherichia coli* infection in intestinal epithelial cells

**DOI:** 10.1111/1750-3841.70079

**Published:** 2025-02-20

**Authors:** Murphy Lam Yim Wan, Vanessa Anna Co, Paul C Turner, Shah P Nagendra, Hani El‐Nezami

**Affiliations:** ^1^ School of Biological Sciences, Faculty of Science, Kadoorie Biological Sciences Building The University of Hong Kong Pokfulam Hong Kong; ^2^ Department of Laboratory Medicine, Division of Microbiology Immunology and Glycobiology, Lund University Lund Sweden; ^3^ School of Medicine, Pharmacy and Biomedical Sciences, Faculty of Science and Health University of Portsmouth Portsmouth UK; ^4^ Maryland Institute for Applied Environmental Health, School of Public Health University of Maryland College Park Maryland USA; ^5^ Institute of Public Health and Clinical Nutrition University of Eastern Finland Kuopio Finland

**Keywords:** bacterial infection, deoxynivalenol, enteroinvasive *Escherichia coli*, inflammatory responses, mucins

## Abstract

**Abstract:**

Deoxynivalenol (DON) is a common mycotoxin in crops that could induce intestinal inflammation, affecting the susceptibility of intestinal epithelial cells (IECs) to pathogen infection. This study aimed to investigate DON's effects on mucin and cytokine production as part of the local immune system and how it affected intestinal susceptibility to pathogen infection. Caco‐2 cells were exposed to DON followed by acute enteroinvasive *Escherichia coli* (EIEC) infection. An increase in EIEC attachment to DON‐exposed cells was observed, probably in part, mediated by secretory MUC5AC mucins and membrane‐bound MUC4 and MUC17 mucins. Additionally, DON with EIEC posttreatment led to significant changes in the gene expression of several proinflammatory cytokines (IL1α, IL1β, IL6, IL8, TNFα, and MCP‐1), which may be in part, mediated by NK‐κB and/or MAPK signaling pathways. These data suggested DON may exert immunomodulatory effects on IECs, altering the IEC susceptibility to bacterial infection.

**Practical Application:**

The results suggested that DON might modulate immune responses by affecting mucus and cytokine production, which may affect the susceptibility of intestinal epithelial cells to pathogen infection.

## 1 INTRODUCTION

The mycotoxin deoxynivalenol (DON), also known as vomitoxin, is a mycotoxin produced as a secondary metabolite by *Fusarium* fungi, particularly *Fusarium graminearum* and *F. culmorum* (Wegulo, 2012). These fungi are commonly found in the soil and can infect cereal crops such as wheat, barley, oats, and maize. Contamination by DON occurs primarily when these fungi infect crops during wet and humid conditions in the field or during improper storage, leading to significant contamination of both human food and animal feed (Neme & Mohammed, 2017). Human ingestion of DON‐contaminated grains can lead to different diseases, including nausea, vomiting, diarrhea, abdominal pain, headache, dizziness, and fever (Awuchi et al., 2021). The toxic effects of DON are of particular concern due to the widespread consumption of grains and cereals, which can lead to significant exposure in both humans and livestock (De Ruyck et al., 2015).

DON has been known to be immunotoxic affecting cells of the immune system and the gastrointestinal tract. DON induces “ribotoxic stress response”, which results in the elevation of many pro‐inflammatory genes such as cyclooxygenase‐2 (COX‐2) and inducible nitric oxide synthase as well as cytokines such as interleukin (IL)‐6, IL‐1β and tumor necrosis factor (TNF)‐α (Moon & Pestka, 2002, 2003; Pestka, 2010; Pestka et al., 2004).

Foodborne pathogens can cause severe diseases including diarrhea, which are of considerable public health concern. The most common foodborne pathogens found to contaminate food include *Escherichia coli* (*E. coli*) spp., *Clostridium perfringens*, *Listeria* spp., and *Salmonella enterica* serotypes Enteritidis (Maciorowski et al., 2007; Scallan et al., 2011). Among them, the most important foodborne pathogen is enteroinvasive *E. coli* (EIEC), which are intracellular pathogens that invade colonic epithelial cells by first penetrating the intestinal epithelium through microfold cells (M cells) to gain access to the submucosa, producing a rather localized infection with subsequent destruction of the underlying mucosa as a result of their active replication (Croxen et al., 2013).

The mucus lining the intestinal epithelium provides an important physicochemical barrier against ingested pathogens and toxins. It is composed of a mixture of mucins, which are heavily glycosylated with O‐linked oligosaccharides and N‐glycan chains linked to protein backbones. There are two different structural and functional classes of mucins: secreted gel‐forming mucins (MUC2, MUC5AC, MUC5B, MUC6, and MUC19) and transmembrane (membrane‐bound) mucins (MUC1, MUC3A, MUC3B, MUC4, MUC12, and MUC17; Grondin et al., 2020). To initiate infection processes, bacteria need to find ways to ensure their survival and colonization by first adhering to the intestinal epithelium (Torres & Kaper, 2003). Mucins can effectively impede the ability of bacteria and viruses to invade and colonize the cells. They also prevent the spread of pathogens along the mucosal surfaces and limit the exposure of mucosal cells to microbial toxins (Liévin‐Le Moal & Servin, 2006; Linden et al., 2008).

Previous studies from our laboratory and others have shown that DON affected mucus production (Antonissen et al., 2015; Pinton et al., 2015; Wan et al., 2014) and also cytokine production (Alassane‐Kpembi et al., 2017; Van De Walle et al., 2010, 2008; Wan et al., 2013; Yang et al., 2019; Ying et al., 2019). Bacterial pathogens such as *E. coli* could also trigger host inflammatory responses by enhancing mucin expression, which helps protect the gut epithelium from bacterial intrusion (Vieira et al., 2010; Xue et al., 2014), and by stimulating cytokine production (Eaves‐Pyles et al., 2008; Jung et al., 1995). *E. coli* is also known to activate the transcription factor nuclear factor κ‐light chain‐enhancer of activated B cells (NF‐κB) and initiate inflammatory responses by producing inflammatory cytokines and chemokines (Dąbek et al., 2010; Underhill & Ozinsky, 2002). The intestinal tract serves as both the primary barrier against ingested mycotoxins and the first line of defense against intestinal bacterial infections. Previous studies have shown that ingestion of some mycotoxins increases the susceptibility to experimental or natural mucosal infections (Fukata et al., 1996; Oswald et al., 2003; Stoev et al., 2000; Tai & Pestka, 1988; Vandenbroucke et al., 2011). So far, there is only one study reporting that DON rendered the intestinal epithelium more susceptible to *Salmonella Typhimurium*, potentiating inflammatory responses in the gut (Vandenbroucke et al., 2011).

Accordingly, it was hypothesized that DON could also increase the susceptibility of intestinal epithelial cells (IECs) to an EIEC infection and trigger intestinal inflammation. With DON and EIEC infections being emerging issues with possible deleterious consequences for both animal and human health and with the gastrointestinal tract being the primary target, this study aimed to investigate the effects of physiologically relevant concentrations of DON on intestinal susceptibility to acute EIEC infection. In our study, we examined the impact of DON and EIEC on mucus production and the gene expression of six specific mucins: MUC1, MUC3, MUC4, MUC5AC, MUC5B, and MUC17. MUC1, MUC3, and MUC4 are transmembrane mucins that contribute to cellular signaling and help limit bacterial adhesion, while MUC5AC and MUC5B are gel‐forming mucins that provide a physical barrier by forming a protective mucus layer. MUC17, another membrane‐bound mucin, plays a similar role in maintaining barrier function and preventing epithelial damage (Grondin et al., 2020). These mucins are prominently expressed in the gastrointestinal tract, making them key players in the body's defense mechanisms against harmful pathogens and toxins. Their involvement in immune responses makes them ideal candidates for studying the effects of bacterial invasion and toxin exposure on gut health (Corfield et al., 2000; Linden et al., 2008). Moreover, since the NF‐κB and MAPK pathways are often involved in the epithelial inflammatory response, both pathways were explored as possible underlying mechanisms. Of the cell line models, the Caco‐2 cell line, originally isolated from human colon adenocarcinoma, despite lack of mucus production, is among the most commonly used in vitro models of the intestinal epithelium to study bacterial adherence and invasion (Ganan et al., 2010; Khodaii et al., 2017; Resta‐Lenert & Barrett, 2003; Resta‐Lenert et al., 2011). Caco‐2 cells were grown at semi‐confluence stages (2 to 3 days of culture) and used in this study, as they were less differentiated, exhibited higher permeability, and were more susceptible to bacterial invasion, making them particularly suitable for studying pathogen–host interactions.

## 2 MATERIALS AND METHODS

### 2.1 Chemicals and reagents

Minimum essential medium (MEM), fetal bovine serum (FBS), and all other cell culture reagents were obtained from Gibco‐Life Technology. DON was obtained from Sigma Chemical Company and was dissolved in dimethyl sulfoxide (DMSO; Sigma) and stored at −20°C before use. In addition, Alcian Blue 8XG solution, triton X‐100, paraformaldehyde, formaldehyde, bovine serum albumin (BSA), phosphate‐buffered saline (PBS), sodium bicarbonate, diethylpyrocarbonate (DEPC) and chloroform were purchased from Sigma. The periodic acid‐schiff (PAS) staining kit was from Atom Scientific Ltd. RNAisoPlus was purchased from Takara. HiScript RT SuperMix for quantitative polymerase chain reaction (qPCR) and AceQ qPCR SYBR Green Master Mix were obtained from Vazyme Biotech Co.

### 2.2 Cell line and culture conditions

Caco‐2 cells obtained from the ATCC (HTB‐37) were maintained at 37°C, 5% CO_2_, and 90% relative humidity in MEM supplemented with 20% FBS. Routinely, cells were subcultured 1–2 times a week using trypsin‐EDTA (0.25%, 0.53 mM) and seeded at a density of 2 × 10^6^ cells per 180 cm^2^ flask. All cells were screened for mycoplasma contamination with a MycoAlert mycoplasma detection kit (Lonza) prior to use.

### 2.3 Bacteria preparation

Enteroinvasive *E. coli* (EIEC O29:NM) was from Prof Wei Chen from the State Key Laboratory of Food Science and Technology, Jiangnan University, or from ATCC (#43892). EIEC were incubated overnight at 37°C in Luria‐Bertani (LB) broth until the stationary phase was reached. Subcultures of the overnight cultures in the fresh medium were grown to a phase of exponential growth. Cells were centrifuged at 4000 rpm (Beckman Coulter; GS‐6R centrifuge) for 5 min, washed with PBS twice, and suspended in MEM supplemented with 20% FBS to desired concentrations before adding to the epithelial cell layers.

### 2.4 Invasion and adhesion assay

The number of cells in each of the 24‐well plates following DON treatment was first determined by trypan blue (Gibco) exclusion assay. Invasion assays were performed using the gentamicin protection assay as described previously (Boudeau et al., 1999).

Briefly, Caoc‐2 cells were seeded in 24‐well plates (1 × 10^5^ cells) and cultured for 2 days. After 2 days, cells were treated with a medium containing exponentially grown EIEC at multiplicities of infection (MOI) ranging from 1:1 to 1000:1 to the cells for 1 or 2 h to optimize the MOI and incubation duration for subsequent experiments. Following optimization, cells were treated with different concentrations of DON (0, 8, or 16 µM) for 24 h. After the 24‐h DON treatment, cells were treated with a medium containing exponentially grown EIEC at a MOI of 250:1. The addition of EIEC had a minimal effect on the pH of the medium (pH 7.0 ± 0.2). This pH level was similar to that of the control treatment and the normal growing conditions for IECs. After 1 h of incubation, cells were washed with PBS three times and incubated in a medium with gentamicin (50 µg/mL) (for cells infected with invasive bacteria or uninfected controls) for another 1 h at 37°C, to kill any extracellular bacteria after the infection period. Following gentamicin treatment, cells were lysed with 0.1% Triton X‐100 (Sigma) in deionized water to release the intracellular bacteria. Samples were diluted and plated onto LB agar plates to determine the colony‐forming unit (CFU) of invading bacteria. In control experiments, gentamicin has no significant effect on any of the parameters measured. Furthermore, no significant bacterial overgrowth was observed over the duration of the experiment under all conditions tested. The percentage of invading bacteria was expressed as CFU_(invading bacteria)_ of infected cells divided by CFU_(total bacteria)_, normalized to the number of cells per well across different concentrations of DON treatment.

To determine the total number of cell‐associated bacteria corresponding to adherent and intracellular bacteria, cells were washed with PBS three times to remove any nonadherent bacteria and lysed after 1 h infection period, and the bacteria were quantified as described above. This step ensured that the bacteria remaining on or within the cells were truly cell‐associated. The number of adhering bacteria was determined by subtracting the number of invading bacteria from the total number of cell‐associated bacteria. The percentage of adhering bacteria was expressed as CFU_(adhering bacteria)_ of infected cells divided by CFU_(total bacteria)_, normalized to the number of cells per well across different concentrations of DON treatment.

### 2.5 Cell viability by CCK‐8 assay

A CCK‐8 colorimetric assay was performed to assess cell viability/cytotoxicity in response to different concentrations of DON without or with EIEC posttreatment. The CCK assay is a colorimetric assay based on the reduction of a tetrazolium salt, WST‐8 (4‐[3‐(2‐methoxy‐4‐nitrophenyl)‐2‐[4‐nitrophenyl]‐2H‐5‐tetrazolio]‐1,3‐benzene disulfonate sodium salt), to a water‐soluble formazan by cellular NADH or NADPH (Ishiyama et al., 1997). The assay was performed following the manufacturer's instructions (Dojindo, Kumamoto, Japan).

In brief, cells were seeded at 2 × 10^4^ cells/well in 96‐well culture plates and allowed to grow for 3 days. At 3 days, cells were treated with different concentrations of DON (0, 2, 4, 8, and 16 µM) for 24 h. After 24‐h DON treatment, Caco‐2 cells were treated with a medium containing exponentially grown EIEC bacteria at a MOI of 250:1 to the cells for 1 h. After that, cells were washed and incubated in a medium with gentamicin (50 µg/mL) for another 1 h. At the end of the incubation, CCK‐8 solution (10 µL) was added to each well, and the cells were incubated at 37°C for 1 h. The color intensity (absorbance) was determined using a microplate reader (model 550, BioRad) at 450 nm. Cell viability was expressed as the percentage of the mean value normalized to the control (untreated cells). For each treatment, the mean value was obtained from at least six wells.

### 2.6 Alcian blue/periodic acid/Schiff mucus staining

Caco‐2 cells were seeded in a 24‐well plate and allowed to grow for 2 days. Cells were then treated with DON (0, 8, 16 µM) for 24 h, followed by treatment with medium containing exponentially grown EIEC bacteria at a MOI of 250:1 to the cells for 1 h. At the end of treatment, the media were carefully removed from the cell cultures, and the cells were fixed in 4% v/v formaldehyde solution for 20 min at room temperature.

To visualize the acidic mucus, the cultures were stained with Alcian blue 8XG solution (1% Alcian blue solution in 3% acetic acid, pH2.5) for 30 min. Excess Alcian blue was then removed by washing the cells with distilled water for 10 min. For detecting neutral mucins, the PAS reagent method was used according to the manufacturer's instructions. The cells were incubated with 1% periodic acid solution at room temperature for 5 min, followed by incubation with Schiff reagent for 20 min. Afterward, the cells were washed with distilled water for 5 min and counterstained with hematoxylin for 1 min. The hematoxylin was subsequently removed by washing the cells with distilled water for 10 min. The stained mucus was observed and imaged using the EVOS microscope (20× objective lens; Life Technologies).

### 2.7 Enzyme‐linked lectin assay for total mucin glycoprotein secretion

The total production of mucins consists of both mucins in the cell lysate and mucins secreted into the cell culture supernatant. Total mucin‐like glycoprotein production was therefore calculated as the sum of mucin proteins present in cell lysate and in cell culture supernatant. Cell lysates and supernatants from cell culture were collected and assayed for total mucin secretion using the enzyme‐linked lectin assay (ELLA) as previously described (Wan et al., 2014, 2018).

Briefly, Caco‐2 cells were seeded onto 96‐well plates and treated or not with DON with or without EIEC infection as described above and were fixed with 4% paraformaldehyde (PFA) for 30 min at room temperature. After fixation, cells were washed two times with 0.05% PBS‐Tween 20 (PBS‐Tw). Cells were blocked for 1 h at room temperature in PBS supplemented with 2% BSA and 0.1% Triton‐X100. Biotinylated‐conjugated wheat germ agglutinin (WGA; 1:10 000 dilution) was then added to the wells for 1 h at room temperature followed by incubation with avidin peroxidase (1:10 000 dilution) for another hour. Cells were washed two times with PBS‐Tw and 3,3′,5,5′‐tetramethylbenzidine peroxidase substrate (BioLegend) was added. The reaction was stopped with 2N sulphuric acid (Merck) and the optical density was read at 490 nm using the Multiskan microplate spectrophotometer (ThermoFisher Scientific). Total mucin‐like glycoprotein production was normalized to the number of cells per well following each treatment. This method was used previously in our laboratory and has been widely employed by other researchers for measuring total mucin‐like glycoprotein in cell lysates and supernatants (Ganan et al., 2010; Hewson et al., 2004; Resta‐Lenert & Barrett, 2003; Resta‐Lenert et al., 2011).

### 2.8 qPCR analysis

Caco‐2 cells were seeded onto 6‐well plates and treated or not with DON with or without EIEC infection as described above. After treatment, total RNA was extracted using RNAiso™ Plus according to the manufacturer's instructions. The concentration of RNA was measured by using NanoDrop ND‐1000 Spectrophotometer (Nano‐Drop Technologies) with purity ascertained by (A260/A280) of >1.8. RNA integrity was checked by running the RNA sample on ∼1% agarose gel. The total RNA (500 ng) from each sample was converted into cDNA using HiScript™ RT SuperMix for qPCR according to the manufacturer's instructions. qPCR was performed to quantify the products of interest, cytokines, and chemokines (IL‐1β, IL‐6, IL‐8, TNF‐α, MCP‐1), mucins (MUC1, MUC3, MUC4, MUC5AC, MUC5B, MUC17), and signaling molecule (NF‐κB). Assessment of glyceraldehyde‐3‐phosphate dehydrogenase (GAPDH) levels was also performed which served as an internal control for RNA integrity and loading. Human‐specific primers were described in Table 1. For analyses on a StepOnePlus™ Real‐Time PCR system (Applied Biosystems), 2 µL of 5X diluted cDNA was added to AceQ qPCR SYBR Green Master Mix, to obtain final primer concentrations of 500 nM/primer in a final volume of 10 µL. The sample was centrifuged briefly and run on the PCR machine using the default fast program (45 cycles of 95°C for 3 s, 60°C for 30 s). To ensure the reliability of qPCR data, the amplicons were kept short (<250 bp) (Nolan et al., 2006). All PCR reactions were performed in duplicate. Negative controls consisting of PCR mix components without cDNA were used for all primers. The relative product levels were quantified using the 2^−△△CT^ method as described previously (Livak & Schmittgen, 2001).

**TABLE 1:** Human‐specific primer sequences for qPCR.

Gene name	Abbreviated name	Product length (bp)	Forward primer sequence (5′–3′)	Reverse primer sequence (5′–3′)	Accession number	Reference
Mucin 1	MUC1	105	GTGGTGGTACAATTGACTCTGG	GTTATATCGAGAGGCTGCTTCC	NM_001204294.1	NM_001204294.1
Mucin 3	MUC3	113	CTGCAACTACCAGCACTTCTTC	TATAGTTCCTGGACAGGGTGTG	NM_005960.2	NM_005960.2
Mucin 4	MUC4	113	AGGCTACCTCAAGACTCACCTC	TCATTCTCCTTGAAGAATCCTG	NM_018406.7	NM_018406.7
Mucin 5AC	MUC5AC	131	CTCCTACCAATGCTCTGTA	GTTGCAGAAGCAGGTTTG	NM_001304359.2	(Wan et al., 2014)
Mucin 5B	MUC5B	154	GACAGAGACGACAATGAG	CCTGATGTTTTCAAAAGTTTC	NM_002458.3	(Wan et al., 2014)
Mucin 17	MUC17	122	GTTTCAACACCACTGGCACC	CTGGTCCCGGTACTCCACTA	NM_001040105.1	NM_001040105.1
Interleukin‐1β	IL1B	138	TGGAGCAACAAGTGGTGTTC	GCTGTAGAGTGGGCTTATCATC	NM_000576.2	NM_000576.2
Interleukin‐6	IL6	100	TGAAAGCAGCAAAGAGGCACT	GCAAGTCTCCTCATTGAATCCAG	NM_000600.5	NM_000600.5
Interleukin‐8	IL8	98	CTGATTTCTGCAGCTCTGTG	GGGTGGAAAGGTTTGGAGTATG	NM_000584.4	NM_000584.4
Tumor necrosis factor‐α	TNFA	93	CTGCTGCACTTTGGAGTGAT	AGATGATCTGACTGCCTGGG	NM_000594.4	NM_000594.4
Monocyte chemoattractant protein 1	CCL3	171	CCCCAGTCACCTGCTGTTAT	TGGAATCCTGAACCCACTTC	NM_002982.4	NM_002982.4
RELA proto‐oncogene, NF‐kB subunit	RELA	112	TCTGCTTCCAGGTGACAGTG	ATCTTGAGCTCGGCAGTGTT	NM_021975.4	(Garg et al., 2013)
Glyceraldehyde 3‐phosphate dehydrogenase	GAPDH	159	CATGTTCGTCATGGGGTGAACCA	AGTGATGGCATGGACTGTGGTCAT	NM_002046.7	NM_002046.7

### 2.9 Protein extraction, SDS‐PAGE, and immunoblotting

The cells, undergoing the same treatment as described for qPCR, were washed with PBS and extracted for total proteins (whole cell extracts). In brief, 100 µL of RIPA lysis buffer, supplemented with a protease inhibitor cocktail (Sigma) and phosphatase inhibitors (Cell Signaling) was used to extract total proteins from the cells. Protein concentration was determined by the DC protein assay (BioRad).

Proteins (10–50 µg) were loaded onto 10% sodium dodecyl sulfate‐polyacrylamide gel (BioRad), separated by electrophoresis (SDS‐PAGE), and then blotted onto a polyvinylidene difluoride membrane (Millipore). The membrane was blocked with 5% BSA in Tris‐buffered saline (TBS) containing 0.05% (v/v) Tween 20 (TBS‐Tw) buffer. Proteins were probed by immunoblotting with diluted rabbit primary antibodies from Cell Signaling (1:1000) for NF‐κB p65 (#8242), p44/42 MAPK (Erk1/2) (#4695), phospho‐p44/42 MAPK (Erk1/2) (#4370), JNK2 (#9258), phospho‐SAPK/JNK (#4668), p38 MAPK (#8690), phospho‐p38 MAPK (#4511), and GAPDH (ab181602, Abcam), followed by horseradish peroxidase (HRP)‐conjugated anti‐rabbit IgG (#170‐6515, BioRad) secondary antibodies. The blots were developed using the Clarity Western ECL blotting kit (BioRad) and chemiluminescence was detected with a digital imaging system (ChemiDoc XRS+ system with image lab software, BioRad). Quantification was performed by Image Lab software version 6.0 (Bio‐Rad) by densitometric analysis (Schneider et al., 2012), with equal‐sized boxes (for each antibody) drawn around bands, and background values taken below each band of interest to account for nonspecific antibody staining in the lane.

### 2.10 Statistical analyses

All assays were expressed as mean ± standard error of mean (SEM) for the number of separate experiments indicated. Data analyses were performed using the GraphPad PRISM 9.0 software (Graphpad Software Inc.). All data were first evaluated for normality with the Shapiro–Wilk and Levene's variance homogeneity test. For parametric data, one‐way analysis of variance (ANOVA) followed by Dunnet's test against a control group; for nonparametric data, one‐way ANOVA with the Kruskal–Wallis test, followed by the Dunn's multiple comparisons test was used to identify significant differences against a control group. Data were considered as significantly different at *p *< 0.05, according to the post hoc ANOVA statistical analysis.

## 3 RESULTS

### 3.1 DON increased EIEC adhesion but reduced invasion

**FIGURE 1:**
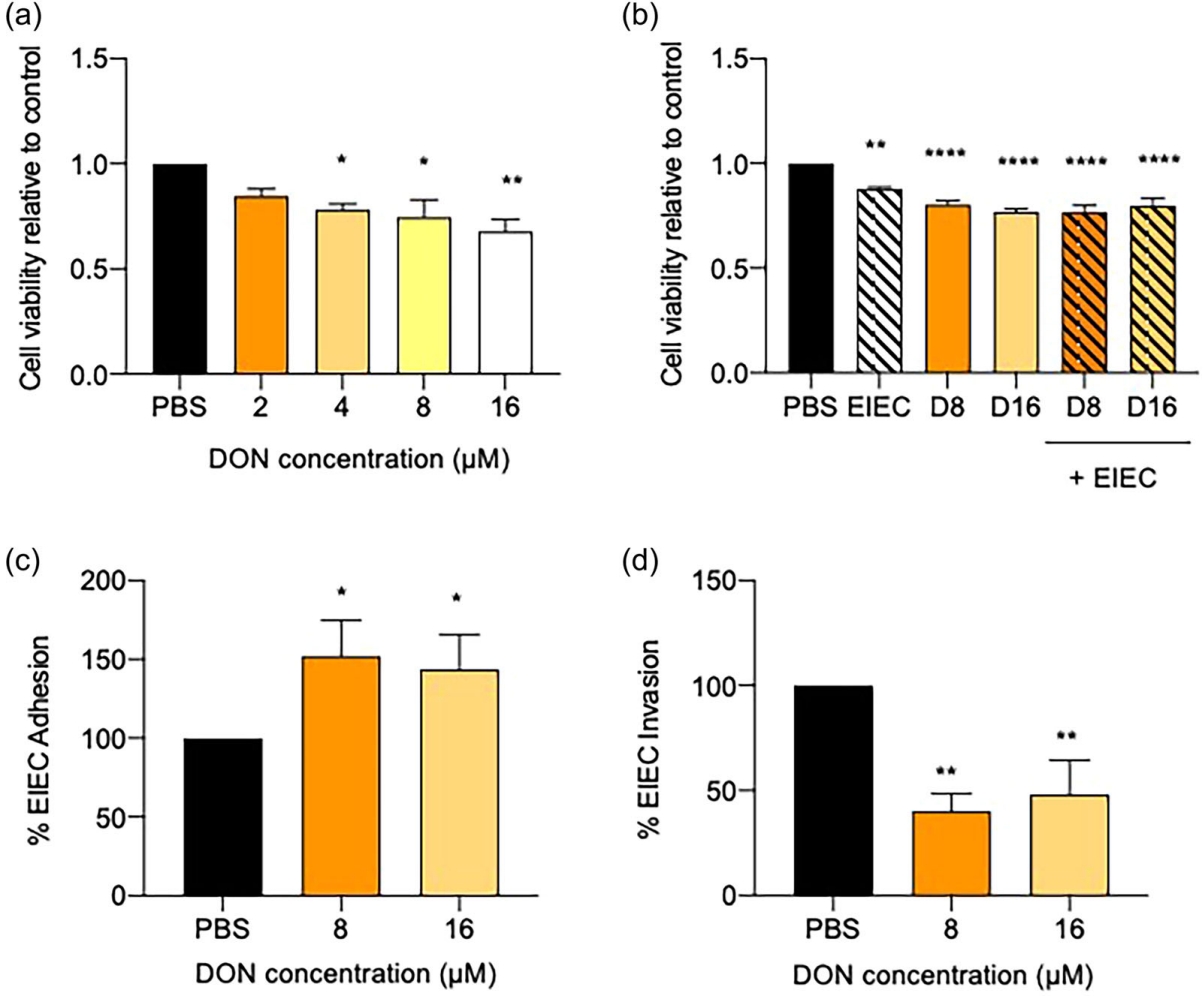
(a, b) Effects of DON without or with EIEC postinfection on cell viability. (a) Preliminary screening of DON concentrations for subsequent experiments. Cell viability data of caco‐2 cells treated with different concentrations of DON (0, 2, 4, 8, and 16 µM) for 24 h. (b) Cell viability data of Caco‐2 cells treated with DON (8 and 16 µM) for 24 h without or with EIEC bacteria posttreatment at a multiplicity of infection (MOI) of 250:1 to the cells for 1 h. Control received appropriate carriers. Results were shown as mean ± SEM, which are from four independent experiments performed in six replicates. *^,^ **^,^ ***^,^ ***p < 0.05, 0.01, 0.001, and 0.0001 compared with PBS control. One‐way ANOVA post‐Dunnet's test. (c, d) Effects of 24 h of DON incubation on Caco‐2 cells without or with EIEC postinfection (1 h) on bacterial adhesion and invasion. The percentage of (c) adhering and (d) invaded bacteria were calculated as described in Materials and Methods. Results were shown as mean of ±SEM, which are from four separate experiments performed in duplicates. *^,^ **p < 0.05 and 0.01 compared with PBS control. One‐way ANOVA post‐Dunn's test.

Since adhesion and invasion are both important processes in bacterial pathogenesis, the effects of DON on EIEC adhesion and invasion were assessed by a modified bacterial adherence assay and gentamicin protection assay (Figure 1). A preliminary bacterial invasion experiment was conducted in our laboratory by adding EIEC at MOI of 1:1, 2.5:1, 10:1, 25:1, 100:1, 250:1, 500:1, 1000:1 to the cells for 1 or 2 h (Figure S1). Results showed that EIEC at MOI of 250:1 for 1 h achieved the highest invasion number, as determined by colony counts. Therefore, the MOI of 250:1 and 1 h of EIEC incubation were chosen for all the subsequent experiments.

Next, to determine the appropriate concentrations of DON used for subsequent experiments, the CCK assay was performed to assess the effect of DON treatment on cell viability (Figure 1a). DON resulted in a significant concentration‐dependent reduction in cell viability at 4, 8, and 16 µM (*p *< 0.05). DON at the concentrations of 8 and 16 µM were selected for the subsequent experimental assays.

The combined effects of DON and EIEC on cell viability, bacterial adhesion, and invasion were then investigated. The addition of EIEC to cells pretreated with 8 and 16 µM DON significantly reduced cell viability compared with control (PBS) (*p *< 0.0001), but the reduction was not significantly different from cells treated with EIEC or DON alone (Figure 1b).

Moreover, DON pre‐treatment at 8 and 16 µM caused a significant increase in EIEC adhesion to the cells (*p *< 0.05) (Figure 1c). However, DON pretreatment significantly reduced EIEC invasion (*p *< 0.05; Figure 1d). These findings suggest that while DON enhanced EIEC adherence, it reduced bacterial invasion. Furthermore, exposure of cells to DON and EIEC, either alone or in combination, led to a significant reduction in cell viability.

### 3.2 DON and EIEC contamination altered mucus production

**FIGURE 2:**
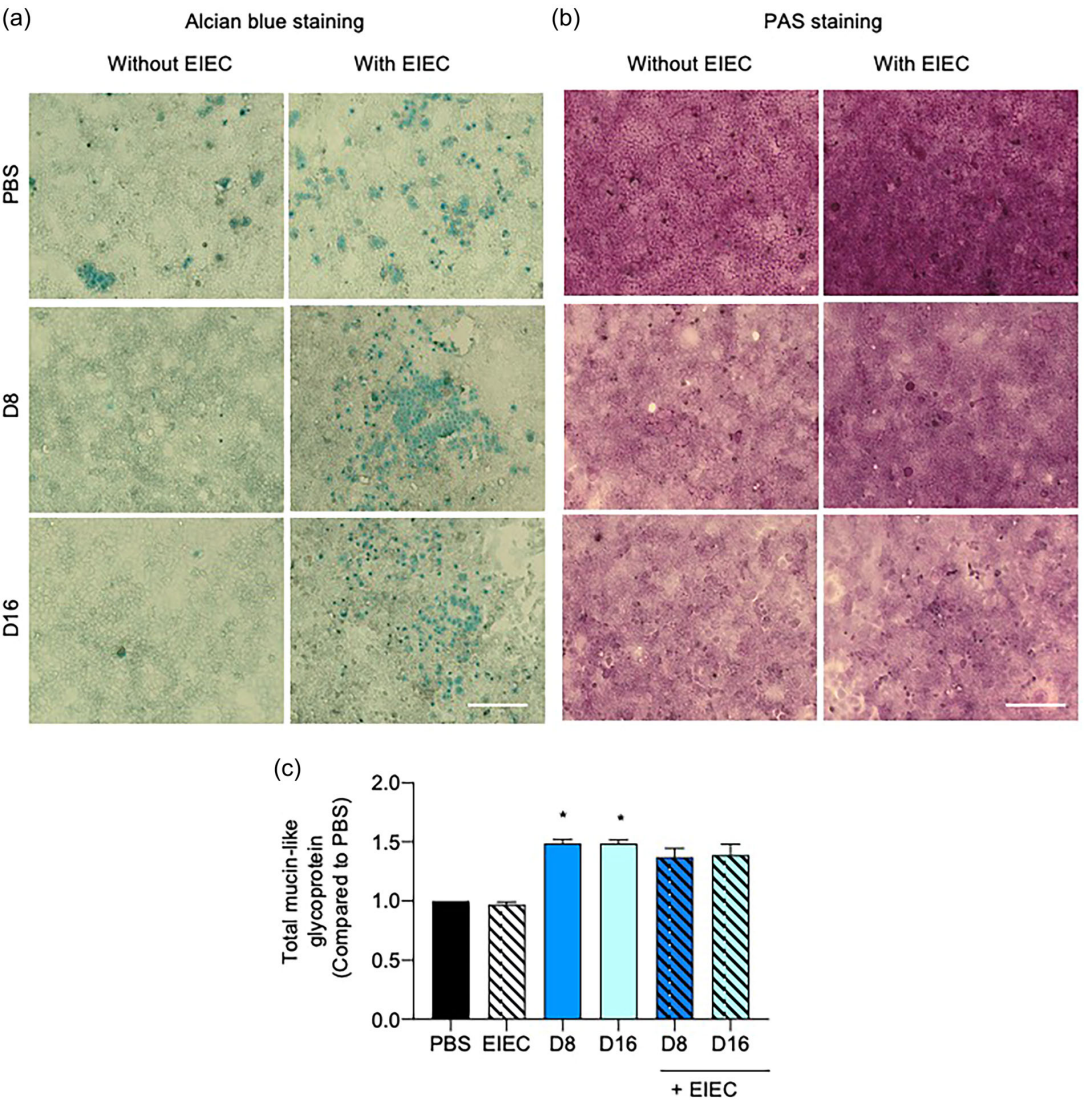
(a, b) Effects of 24 h DON incubation without or with EIEC postinfection (1 h) on mucus production visualized by Alcian blue/PAS staining. (a) Alcian blue staining of Caco‐2 cells treated with DON for 24 h without or with EIEC postinfection (1 h). Blue areas indicate acidic mucin deposition (20× objective lens). Scale bar = 200 µm. (b) PAS staining of Caco‐2 cells treated with DON for 24 h without or with EIEC postinfection (1 h). Fuchsia areas indicate neutral mucin deposition (20× objective lens). Scale bar = 200 µm. (c) Effects of 24 h DON incubation without or with EIEC postinfection (1 h) on mucin‐like glycoprotein production as measured by the enzyme‐linked lectin assay (ELLA). Results were shown as mean of ±SEM from six independent experiments. *p < 0.05 compared with PBS control. One‐way ANOVA post‐Dunn's test.

Mucin production is a critical component of intestinal mucus, forming a physical barrier that protects against bacterial infection. The effects of DON and EIEC exposure on mucus production were visualized by Alcian blue/PAS staining, and total mucin‐like glycoprotein secretion—both intracellular (from cell lysates) and extracellular (from culture supernatants) were measured and analyzed using the ELLA (Figure 2).

The levels of Alcian blue/PAS staining were lower in cells treated with DON alone, but higher in cells treated with EIEC alone, compared with the PBS control. The addition of EIEC further increased the levels of staining, compared with their pathogen‐free counterparts (Figure 2a,b). With regards to total mucin‐like glycoprotein secretion, treatment with 8 and 16 µM DON alone increased mucin‐like glycoprotein production significantly (*p *< 0.05). However, the addition of EIEC did not cause any significant change to the amount of mucin‐like glycoprotein produced compared with their pathogen‐free counterparts (Figure 2c).

**FIGURE 3:**
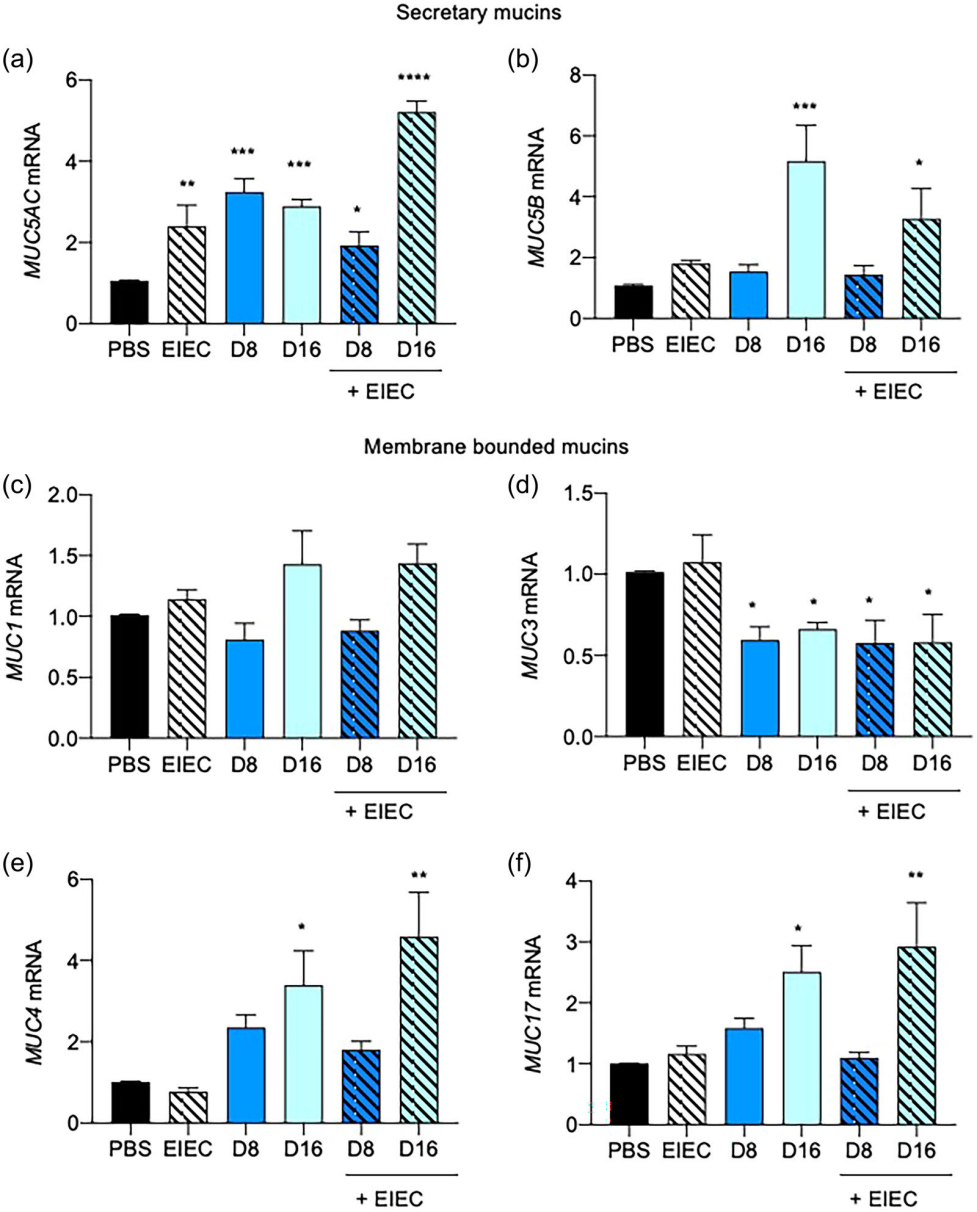
(a–f) Effects of 24 h DON incubation without or with EIEC postinfection (1 h) on mucin (*MUC*) gene expression. (a, b) Secretory *MUC5AC* and *MUC5B*, and (c–f) membrane‐bound *MUC1, MUC3, MUC4*, and *MUC17* mRNA expression was measured by qPCR, with *GAPDH* as the internal control. Results were shown as mean of ±SEM from five independent experiments. *^,^ **^,^ *** *p *< 0.05, 0.01, and 0.001 compared with PBS control. One‐way ANOVA post‐Dunnet's test.

qPCR was performed to assess the effects of DON and/or EIEC on mucin mRNA expression. Significant upregulation of *MUC5AC* mRNA was observed when cells were treated with either DON or EIEC alone, and the addition of EIEC further increased *MUC5AC* mRNA levels (Figure 3a). Similarly, significant up‐regulation of *MUC5B* mRNA was observed when cells were treated with 16 µM DON alone, but no further change in *MUC5B* mRNA levels was found with EIEC postinfection (Figure 3b).

*MUC1*, *MUC4*, and *MUC17* mRNA expression increased gradually with increasing DON concentrations (Figure 3c,e,f). *MUC1* mRNA expression remained unchanged in cells treated with DON, with and without EIEC posttreatment, compared with the PBS control. DON pretreatment significantly lowered *MUC3* mRNA expression (*p *< 0.05), but this was not observed in EIEC‐treated cells. Addition of EIEC after DON treatment did not affect *MUC3* mRNA expressions (Figure 3d). For *MUC4* and *MUC17*, treatment of cells with 16 µM DON significantly increased their mRNA levels (*p *< 0.05) compared with the PBS control. Addition of EIEC further increased *MUC4* and *MUC17* mRNA expression (*p *< 0.01; Figure 3e,f).

### 3.3 DON and EIEC modulated proinflammatory cytokine and chemokine gene expression

**FIGURE 4:**
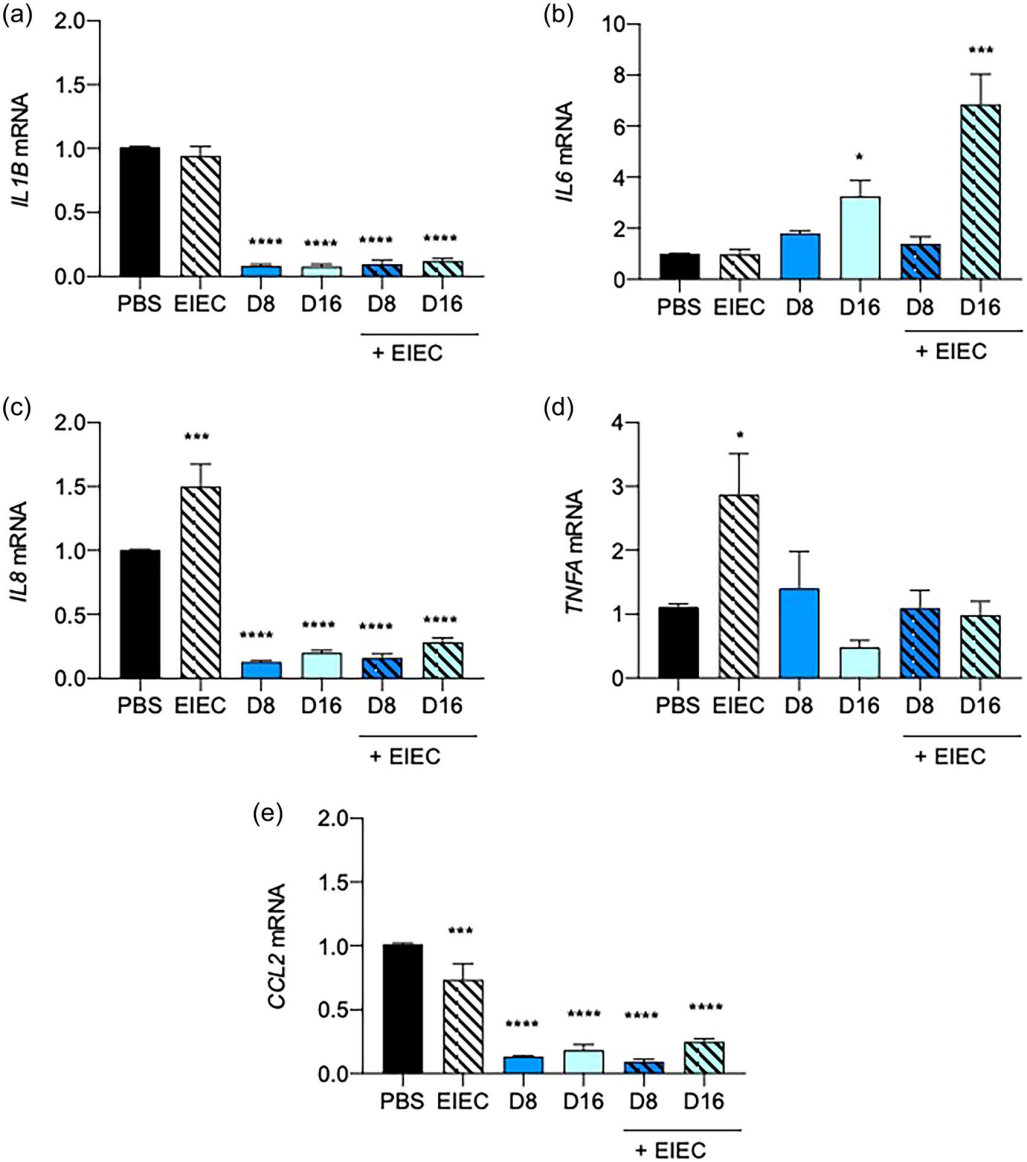
(a–e) Effects of 24 h DON incubation without or with EIEC postinfection (1 h) on cytokine and chemokine gene expression. *IL1B*, *IL6, IL8*, *TNFA*, and *CCL2* mRNA expression were measured by qPCR, with *GAPDH* as the internal control. Results were shown as mean of ±SEM from five independent experiments. *^,^ ***^,^ ****p < 0.05, 0.001, and 0.0001 compared with PBS control. One‐way ANOVA post‐Dunn's test.

Intestinal cytokines regulate innate immune pathways and cellular processes within the gut mucosa. Disruption of these processes or alterations in the cytokine environment can lead to inappropriate inflammation characteristic of conditions, thereby increasing the susceptibility to bacterial infection (Elshaer & Begun, 2017). The effects of treating Caco‐2 culture with DON and EIEC on pro‐inflammatory cytokine and chemokine mRNA expression were examined by qPCR (Figure 4). Treatment with DON, both with and without subsequent EIEC infection, significantly downregulated *IL1B* gene expression (*p *< 0.0001; Figure 4a). Pretreatment with 16 µM DON significantly upregulated *IL6* mRNA expression (*p *< 0.05), and the addition of EIEC further increased the *IL6* mRNA levels (*p *< 0.001; Figure 4b). In the absence of DON, EIEC infection alone significantly increased *IL8* and *TNFA* mRNA expressions (*p *< 0.001 and 0.05, respectively; Figure 4c,d). Pretreatment with DON significantly lowered the *IL8* expression (*p *< 0.0001), but no significant change in *TNFA* mRNA levels was observed. EIEC posttreatment did not cause any further change in *IL8* and *TNFA* mRNA levels. Treatment with DON or EIEC alone significantly downregulated *CCL2* (MCP‐1) mRNA expression (*p *< 0.001), with DON demonstrating a greater ability to modulate *CCL2* mRNA expression than EIEC (*p *< 0.0001). No significant change in *CCL2* mRNA was observed in DON‐treated cells with EIEC posttreatment (Figure 4e).

### 3.4 DON and EIEC activate the NF‐κB p65 signaling pathway

**FIGURE 5:**
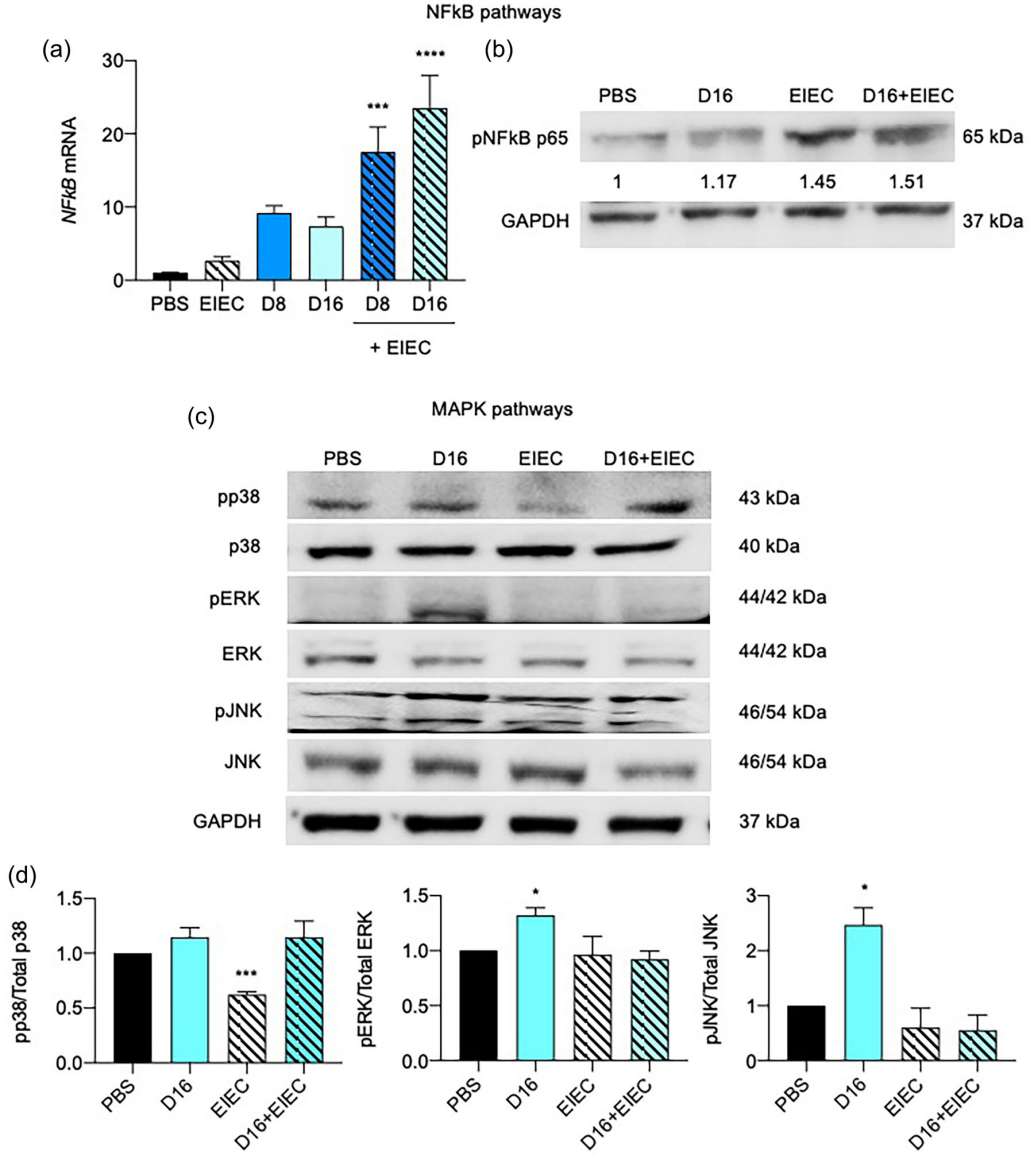
(a–c) Effects of 24 h DON incubation without or with EIEC postinfection (1 h) on NF‐κB and MAPK signaling pathways. (a) *RELA* mRNA expression was measured by qPCR, with *GAPDH* as the internal control. Results were shown as mean of ±SEM from six independent experiments. (b) NF‐κB p65 protein expression was measured by Western blotting, with GAPDH as the internal control. Representative photos of western blotting of NF‐κB p65 and GAPDH. Quantification of Western blot compared with PBS control from three independent experiments. Inset shows group means. (c) Protein samples were also analyzed by Western blot with phospho‐p38, JNK, and ERK antibodies. The total MAPK levels were used as an internal control. Representative photos of western blotting of MAPKs and GAPDH. Results were shown as mean of ±SEM from four independent experiments. *^,^ ***^,^ ****p < 0.05, 0.001, and 0.0001 compared with PBS control. One‐way ANOVA post‐Dunn's test.

p65 (RELA) and p50 (NF‐κB1) are the most commonly found heterodimer complex of NF‐κB, which participate in nuclear translocation and activation of NF‐κB to regulate gene expression and major cellular functions (Garg et al., 2013). Therefore, in our study, qPCR and western blot analyses were used to quantify *RELA* (or NF‐κB p65) levels as an indicator for NF‐κB activation in response to DON and EIEC exposure (Figure 5). Treatment with DON alone resulted in an ascending trend in *RELA* mRNA expression. Addition of EIEC further increased the mRNA expression (*p *< 0.01; Figure 5a). Results obtained from Western blot also revealed similar patterns for NF‐κB protein expression. Upregulation of NF‐κB p65 protein expression was observed in cells infected with EIEC without and with DON pretreatment (Figure 5b).

### 3.5 EIEC inhibited DON‐induced MAPK signaling pathway activation

Previous studies have shown the involvement of MAPK signaling pathways in DON‐induced inflammation in IECs (Van De Walle et al., 2010, 2008). In this study, the three classical MAPK signaling pathways, JNK, p38 MAPK, and extracellular signal‐regulated kinase (ERK) were investigated in DON‐treated cells with and without EIEC postinfection (Figure 5c,d). Western blot data showed that DON treatment alone significantly increased the levels of phosphorylated ERK and JNK protein, indicating activation of these pathways. In contrast, the phosphorylated p38 protein level remained unaffected by DON treatment. Interestingly, the addition of EIEC to DON‐treated cells inhibited the phosphorylation of ERK and JNK proteins. Furthermore, EIEC alone significantly inhibited p38 signaling pathways, however, no change in p38 phosphorylation protein level was observed in cells pretreated with DON.

## 4 DISCUSSION

The present study was the first to investigate the effects of low and relevant concentrations of DON on intestinal susceptibility to acute EIEC infection. The effects of DON and EIEC contamination on mucin, cytokines/chemokines, and related signal transduction pathways were examined in the IECs as part of the local immune system. The concentrations of DON are in accordance with the levels probably encountered in the gastrointestinal tract of animals or human tissues after consumption of food or feed contaminated with DON (Sergent et al., 2006). Assuming that DON ingested in one meal is diluted in 1 L of gastrointestinal fluid and is totally bio‐accessible, the in vitro concentrations to be used in this study correspond to food contamination ranging from 1.18 to 4.72 mg/kg of DON (Van De Walle et al., 2008). The infection protocol (MOI and EIEC treatment duration) was established based on a preliminary experiment in our laboratory. Similar infection protocols were also adopted by other investigators (Ganan et al., 2010; Hewson et al., 2004; Resta‐Lenert & Barrett, 2003; Resta‐Lenert et al., 2011).

Interestingly, reduced EIEC invasion in DON‐pretreated cells did not significantly improve cell viability. This observation may initially seem counterintuitive, as both EIEC and DON are independently known to reduce cell viability. One explanation could be that while DON pretreatment reduced EIEC's ability to invade IECs, this reduction did not directly translate to enhanced cell viability. DON's cytotoxic effects, such as protein synthesis disruption, oxidative stress induction, and activation of apoptotic pathways, may overshadow the benefits of reduced bacterial invasion. In this scenario, the harmful effects of DON could dominate, resulting in cell damage or stress even with fewer invading bacteria. Yet, while bacterial invasion may be reduced, the combined stress from DON toxicity and residual bacterial presence could still lead to significant cell viability loss. Further studies are warranted to elucidate the complex mechanisms through which DON influences both bacterial invasion and cell viability.

Bacterial adherence to host cells is the initial crucial step toward colonization and establishment of infection within the host (Torres & Kaper, 2003). In the present study, DON increased the adhesion of EIEC on IECs but caused a reduced invasion of IECs. This may be attributed to the induction of mucin gene and protein expression in IECs as demonstrated in our study. Indeed, numerous studies have shown altered mucin expression in chronic intestinal inflammatory diseases and cancer, both in animal models and patient cohorts (Heazlewood et al., 2008; Ho et al., 2006, 1993; Larsson et al., 2011; Longman et al., 2006; Moehle et al., 2006; Reis et al., 1999). Both secretory and membrane‐bound mucins are important constituents of the physicochemical barrier for the protection of the epithelial cell surface against undesirable harmful pathogens (Liévin‐Le Moal & Servin, 2006). Overexpression and hypersecretion of the secretory mucins, in particular, MUC5AC and MUC5B are two of the important characteristics of the inflammatory process in mucosa. Previous studies conducted by our laboratory indicate the modulation of biosynthesis of MUC5AC and MUC5B following exposure to DON in differentiated Caco‐2 cells (Wan et al., 2014). However, no data are available concerning the effects of DON and EIEC on mucin production. Our finding demonstrated that MUC5AC and MUC5B mRNA were significantly increased upon DON and EIEC treatment. The rapid elevation of secretory mucin in responses to xenobiotics and bacterial infection is crucial for protecting the intestine against pathogens and its metabolites (Liévin‐Le Moal & Servin, 2006; Melhem et al., 2021).

Furthermore, there is increasing evidence for the role of membrane‐bound mucins in maintaining intestinal mucosal integrity. Among all identified membrane‐bound mucins, MUC3 and MUC17 are the membrane‐bound mucins that are moderately expressed in the colon (Hattrup & Gendler, 2008) and abundantly in both goblet cells and enterocytes of the small intestine (Ho et al., 1993; Kim & Ho, 2010). On the other hand, MUC1 and MUC4 are also expressed in normal intestinal tissues, but they are markedly upregulated in response to bacterial infection (Lindén et al., 2008; McAuley et al., 2007). In this study, we have shown a significant induction in *MUC1*, *MUC4*, and *MUC17* but not *MUC3* mRNA in cells with DON and EIEC co‐exposure. This was in agreement with a previous study that also demonstrated the protective role of MUC17 in the protection of the intestinal mucosa against an EIEC strain (Resta‐Lenert et al., 2011). MUC17 also contributes significantly to maintaining cell homeostasis and modulating chronic inflammatory responses by activating signaling pathways associated with inflammation and cancer. It was postulated that NF‐κB contributes, at least partly to the mucin regulation because all intestinally expressed mucin genes contain a potential or experimentally proven binding site for NF‐κB (Moehle et al., 2006).

NF‐κB transcription factor plays a critical role in the regulation of immune, inflammatory, and acute phase responses and is also implicated in the control of cell proliferation and programmed cell death (Aggarwal et al., 2004). The predominant form of NF‐κB is composed of p65 and p50 subunits. NF‐κB is normally sequestered in the cytoplasm of nonstimulated cells by the inhibitors of NF‐κB (IκB), in which IκBα is the principal subunit. The activation of NF‐κB typically involves the phosphorylation of IκB, followed by IκB ubiquitination and proteome degradation. This releases NF‐κB and allows it to translocate freely to the nucleus in a phosphorylated form to activate gene transcription (Karin & Ben‐Neriah, 2000; Perkins, 2007). NF‐κB is, however, regarded as a potential pathogenic factor that is harmful to the host when excessively or improperly activated (Wullaert et al., 2011). The ability of DON to influence NF‐κB activation has been extensively reported (Adesso et al., 2017; Del Regno et al., 2015; Kalaiselvi et al., 2013; Krishnaswamy et al., 2010; Van De Walle et al., 2008). In this study, we reported that DON increased NF‐κB activation during inflammation. EIEC treatment for 1 h after DON treatment caused a higher up‐regulation of *RELA* mRNA and NF‐κB protein. But of course, NF‐κB is not the only transcriptional regulator influencing mucin expression. Further studies are necessary to understand the mechanisms controlling the expression of mucin.

MAPK signaling pathways, including JNK, p38 MAPK, and ERK, play important roles in activating NF‐κB. It has been suggested that the elevated phosphorylation of the key MAPK proteins may regulate inflammation by influencing pro‐inflammatory cytokine production (Van De Walle et al., 2010, 2008). To determine whether MAPK signaling pathways were involved in the immune responses in cells upon DON and EIEC treatment, the three MAPKs (JNK, p38 MAPK, and ERK) were investigated in this study. Consistent with other previous studies, DON induced phosphorylation of JNK and ERK proteins (Bae & Pestka, 2008; Springler et al., 2016; Yu et al., 2021). The addition of EIEC to DON‐pretreated cells suppressed DON‐induced phosphorylated JNK and ERK protein levels. DON alone did not increase the phosphorylated p38 MAPK protein level, but EIEC alone inhibited the phosphorylated p38 MAPK protein level. Although it is evident that MAPK plays an important role in immune responses to *E. coli* infection (Wang et al., 2007; Zhuang et al., 2017), its role in the adherence and internalization of bacteria into IECs was unclear. It is postulated that such deactivation of MAPK pathways may counteract the adhesion and invasion of bacteria into the cells, which are the major contributing factors to intestinal infection and inflammation (Liu et al., 2012).

To elucidate the proinflammatory mechanisms of IECs in response to DON exposure and EIEC infection, the transcriptional levels of a specific array of the six pro‐inflammatory cytokines and chemokines, IL1α, IL1β, IL6, IL8, TNFα, and MCP‐1 was investigated in the present study. We found that EIEC alone induced the mRNA expression of *IL8* and *TNFA*. Derangement of cytokine/ chemokine production by bacterial infection can lead to chronic inflammatory conditions (Karin et al., 2006; Medzhitov, 2008). However, it was surprising to show that DON treatment significantly downregulated the mRNA expression of *IL1B, IL8*, and *TNFA*. This result was in agreement with a previous report by Ghareeb et al. (2013), which found that in broiler chickens, chronic administration of DON for 5 weeks resulted in significant downregulation of certain cytokines, such as *IFNG* and *IL1B* mRNA in jejunal tissues. Similar suppression of splenic *IFNG* and *IL1B* mRNA was also observed in another study in pigs following DON exposure (Cheng et al., 2006). DON is known to either suppress or stimulate immunological responses, depending on the dose, time, and duration of exposure (Ghareeb et al., 2013). In this context, it becomes evident that DON has a modulating effect on the innate immune response. DON could modify the gene expression of cytokines/ chemokines and thus may affect the susceptibility of humans and animals to disease. Despite the lack of quantifying the levels of proteins that are actually translated from the observed mRNA transcripts, this study is the first to present significant modulation of different pro‐inflammatory cytokine and chemokine mRNA expression in IECs following DON and EIEC exposure, which might merit further investigation of the mechanisms in relation to the functional relevance of mRNA expression such as by determining the protein levels of the selected pro‐inflammatory cytokines by utilizing the quantitative sandwich enzyme immunoassay (ELISA).

## 5 CONCLUSION

The present study suggested the potential involvement in mucins such as secretory MUC5AC mucins and membrane‐bound MUC4 and MUC17 mucins in modifying the attachment and invasion of EIEC and thus affecting the susceptibility to EIEC infection. IECs are also able to express and produce important mediators of inflammation such as cytokines and chemokines that are important for host defense and bacterial recognition. The augmented mucin production and inflammatory stimulation might be a consequence of the activation of the NF‐κB signaling pathway. DON exposure also activated the MAPK signaling molecules, including ERK and JNK through phosphorylation. However, the addition of EIEC to DON pre‐treated cells inhibited the MAPK signaling pathway which might help protect IECs from further damages caused by bacterial infection. Nevertheless, further studies are necessary to examine different bacterial infection scenarios and to identify the complex mechanism(s) by which this mycotoxin acts on the intestinal tract to modulate invasion and colonization by opportunistic pathogens by using molecular approaches, such as high‐throughput mRNA sequencing and proteomics. Epidemiological studies are also needed to assess the extent to which DON are involved in the development of infectious diseases in humans.

## AUTHOR CONTRIBUTIONS

**Murphy Lam Yim Wan**: Conceptualization; methodology; data curation; investigation; formal analysis; visualization; writing—original draft; writing—review and editing; project administration; funding acquisition. **Vanessa Anna Co**: Data curation; investigation; formal analysis; visualization; writing—original draft; writing—review and editing. **Paul C Turner**: Writing—review and editing; visualization; supervision. **Shah P. Nagendra**: Methodology; writing—review and editing. **Hani El‐Nezami**: Conceptualization; investigation; supervision; writing—review and editing; resources; funding acquisition.

## CONFLICT OF INTEREST STATEMENT

The authors declare no conflict of interest.

## Supporting information



Supplementary Fig. 1. Optimization of multiplicities of infection (MOI) and incubation duration for EIEC treatment. Caco‐2 cells were treated with medium containing exponentially grown EIEC at MOIs ranging from 1:1 to 1000:1 for 1 or 2 h. Results showed that EIEC at MOI of 250:1 for 1 h achieved the highest invasion number, as determined by colony counts. These conditions were selected for all subsequent experiments.
