# Supplementary figures and images for: Deoxynivalenol modulated mucin expression and proinflammatory cytokine production, affecting susceptibility to enteroinvasive Escherichia coli infection in intestinal epithelial cells
Source: J Food Sci. 2025 Feb 20;90(2):e70079. doi: 10.1111/1750-3841.70079 (PMC11842951; doi:10.1111/1750-3841.70079)

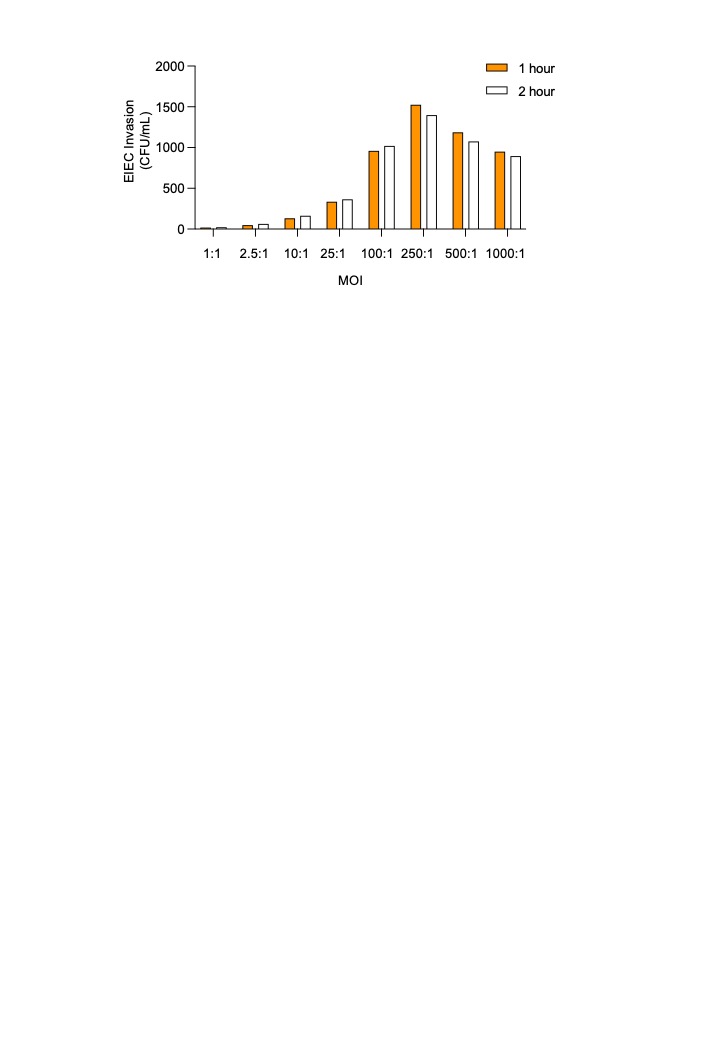

Supplement: Supplementary file 1 — Supplementary Fig. 1. Optimization of multiplicities of infection (MOI) and incubation duration for EIEC treatment. Caco‐2 cells were treated with medium containing exponentially grown EIEC at MOIs ranging from 1:1 to 1000:1 for 1 or 2 h. Results showed that EIEC at MOI of 250:1 for 1 h achieved the highest invasion number, as determined by colony counts. These conditions were selected for all subsequent experiments. [file JFDS-90-0-s001.jpg]
